# Evaluation of an *O*2-Substituted (1–3)-β-D-Glucan, Produced by *Pediococcus parvulus* 2.6, in *ex vivo* Models of Crohn’s Disease

**DOI:** 10.3389/fmicb.2021.621280

**Published:** 2021-02-05

**Authors:** Sara Notararigo, Encarnación Varela, Anna Otal, Iván Cristobo, María Antolín, Francisco Guarner, Alicia Prieto, Paloma López

**Affiliations:** ^1^Department of Microbial: and Plant Biotechnology, Margarita Salas Biological Research Centre (CIB-Margarita Salas-CSIC), Madrid, Spain; ^2^Department of Gastroenterology, Digestive System Research Unit, Institut de Recerca Vall d’Hebron (VHIR), University Hospital Vall d’Hebron, Universitat Autònoma de Barcelona, Barcelona, Spain; ^3^Foundation Health Research Institute of Santiago de Compostela (FIDIS), Santiago de Compostela, Spain; ^4^CIBERehd, Instituto de Salud Carlos III, Madrid, Spain

**Keywords:** bacterial exopolysaccharides, O2-substituted-(1-3)-β-D-glucan, lactic acid bacteria, Immunomodulation, Crohn´s disease anti-inflammatory effect

## Abstract

1,3-β-glucans are extracellular polysaccharides synthesized by microorganisms and plants, with therapeutic potential. Among them, the *O*2-substituted-(1–3)-β-D-glucan, synthesized by some lactic acid bacteria (LAB), has a prebiotic effect on probiotic strains, an immunomodulatory effect on monocyte-derived macrophages, and potentiates the ability of the producer strain to adhere to Caco-2 cells differentiated to enterocytes. In this work, the *O*2-substituted-(1–3)-β-D-glucan polymers produced by GTF glycoyltransferase in the natural host *Pediococcus parvulus* 2.6 and in the recombinant strain *Lactococcus lactis* NZ9000[pNGTF] were tested. Their immunomodulatory activity was investigated in an *ex vivo* model using human biopsies from patients affected by Crohn’s disease (CD). Both polymers had an anti-inflammatory effect including, a reduction of Interleukine 8 both at the level of its gene expression and its secreted levels. The overall data indicate that the *O*2-substituted-(1–3)-β-D-glucan have a potential role in ameliorating inflammation *via* the gut immune system cell modulation.

## Introduction

The exopolysaccharides (EPS) are large, lineal, or branched, extracellular carbohydrate polymers, produced by algae, plants, and bacteria. They are commonly used as food additive due to their rheological properties (bio-thickener, gelling, or viscosifier agents) in the food industry. The demand for new polymers in the food industry is positioning EPS from lactic acid bacteria (LAB), as the new generation of food thickeners. Due to their Generally Regarded As Safe (GRAS) status, they are suitable for the production of fermented and/or functional products. The EPS synthesized by LAB are divided into homopolysaccharides, if they contain only one monosaccharide type or heteropolysaccharides, if they contain various monosaccharides types (Werning et al., 2012). These EPS are known to support bacterial growth, as well as participate in cellular recognition and interaction, surface adhesion, and biofilm formation. Currently, EPS are gaining interest as prebiotics, as modulators of the host immune system (Schmid et al., 2015), and as antiviral agents (Nácher-Vázquez et al., 2015). Their immunomodulatory properties are dependent on their ability to form a suitable tertiary structure. For instance, (1–3)-β-D-glucans (β-glucans), linear or branched in position *O*-4 and *O*-6, are gaining interest as therapeutic targets; various studies have shown their positive influence in reduction of human serum cholesterol levels and their stimulation of the human immune system (Chan et al., 2009; Jin et al., 2018), as well as their potential anti-carcinogenic properties (Chan et al., 2009; Ali et al., 2015).

Recent findings documented that (1–3)-β-glucans are capable of regulating the inflammatory response, modulate immune system cell types (such as peripheral blood immune system cells, intestinal epithelium, and mammalian microglia), through their interaction with membrane receptors, including toll-like receptors, Dectin-1, SIGNR1, complement receptor 3 (CR3), LaCer, and Scavenger, which are differentially expressed in these cells types (Li et al., 2019). Their interaction with a receptor leads to downstream events including the activation of immune system cells, both innate (e.g., macrophages, monocytes, or neutrophils) and adaptive (e.g., T cells or B cells). These interactions may result in differential cytokine production [such as tumor necrosis factor-α (TNF-α), IL-10, IL-8, or IL-12], through the modulation of the nuclear factor kappa-light-chain-enhancer of activate B cells (NF*κ*B) transcription factor, belonging to the mitogen-activated protein kinase (MAPK) pathway (Chan et al., 2009; Volman et al., 2010).

Moreover, it has been reported that the EPS are metabolized in the gut due to the presence of the glycolytic enzyme pools of the microbiota. The exact nature of this process depends on the molecular weight and biochemical composition of the EPS (Bodera, 2008; Kau et al., 2011; Ballesteros Pomar and Gonzalez Arnaiz, 2018). Finally, this hydrolysis is beneficial to both the host and the microbiota itself, because it generates secondary metabolites with the potential to act as probiotic or postbiotic immunomodulators, that ultimately promote and/or restore a healthy environment for the microbiota (Iweala and Nagler, 2019). Laminarin, an *O*6-substituted-(1–3)-β-D-glucan isolated from brown algae, causes a reduction in the expression of pro-inflammatory cytokines, such as IL-6 and IL-1β in pig intestinal mucosa (Heim et al., 2014; Walsh et al., 2015) and counteracts dysbiosis of the microbiota (Rattigan et al., 2020). Moreover, studies performed with human fecal microbiota and commercial laminarin from *Laminaria digitata*, or crude polysaccharide-rich extracts from this algae, indicated that the polysaccharide influences mucus and gut microbiota composition, which results in a potentially beneficial production of short-chain fatty acids (such as butyrate; Devillé et al., 2007; Strain et al., 2020).

Inflammatory bowel disease (IBD) has a multifactorial etiology and includes Crohn’s disease (CD) and ulcerative colitis (UC; Kamada et al., 2013; Morhardt et al., 2019). They are chronic gastrointestinal disorders, where the dysregulation of the immune system is responsible for immunological imbalance characterized by the production of pro-inflammatory cytokines (such as TNF-*α*, INF-*γ*, IL-17, and IL12) into the gut lumen, alteration of microbiota, and the intestinal mucosal barrier (Knights et al., 2013). High inflammation is responsible for abdominal pain, bloody stools, weight loss, diarrhea, etc.

To evaluate therapeutic treatments, current chemically induced animal models of IBD, including sodium dextran-sulfate treatment, resembles UC symptoms, but emulates only partially the inflammatory process that occurs in human CD (Chassaing et al., 2014). However, an *ex vivo* model, using CD mucosal tissue, allows the investigation of how to modulate the inflammation at the gut mucosa level. Previous studies, using this model, have established that certain probiotic bacteria are capable of interacting with immunocompetent cells using the mucosal interface, and thus can modulate locally the production of pro-inflammatory cytokines by inflamed tissue (Borruel et al., 2003; Llopis et al., 2009; Hidalgo-Cantabrana et al., 2015).

In this context, recently, new therapeutic strategies involving the use of probiotic strains have been developed to ameliorate CD patient’s symptoms. Some of these include microorganisms, such as *Lacticaseibacillus rhamnosus* GG, *Limosilactobacillus reuteri*, *Lactobacillus acidophilus*, *Bifidobacterium infantis*, *Saccharomyces boulardii*, *Escherichia coli* Nissle 1917, and *Clostridium butyricum* MIYAIRI 588 (Tsai et al., 2019), which contribute to reduction of inflammation, due to their ability to reduce pathogen adhesion to the intestinal epithelium (blocking their binding site), and by production of antibacterial substances or EPS (Orel and Kamhi Trop, 2014; Oka and Sartor, 2020). In addition, probiotic strains modify the release of cytokines in the intestinal epithelium and inhibit, in the immune system cells, the production of the transcription factor NFkB, leading as a consequence to a reduction of intestinal inflammation (Basson et al., 2017; Oka and Sartor, 2020). In this general context, some probiotic bacteria produce a *O*2-substituted-(1–3)-*β*-D-glucan with prebiotic activity and that stimulate the growth of probiotic LAB (Russo et al., 2011; Pérez-Ramos et al., 2017). Therefore, the producing bacteria or their EPS have potential as adjuvants in the treatment of IBD. Such a role is plausible, taking into account that β-glucans play an important role in the modulation of both the innate immune response, through interaction with dendritic cells (DCs) and macrophages, and also in the adaptive immune response, increasing proliferation of T- and natural killer cells, *via* cytokine release (Bodera, 2008; Zhang et al., 2015).

Only *Pediococcus*, *Lactobacilli*, and *Oenococcus* strains isolated from alcoholic beverages synthesize the *O*2-substituted-(1–3)-β-D-glucan (reviewed in Llamas-Arriba et al., 2019). Moreover, studies performed with *Pediococcus parvulus* 2.6 isolated from cider and with the recombinant *L. lactis* NZ9000[pGTF] strain carrying the pediococcal *gtf* gene, demonstrated that this gene encodes the GTF glycosyltransferase, which catalyzes the synthesis of the *O*2-substituted-(1–3)-β-D-glucan in LAB (Werning et al., 2006, 2008, 2012). Moreover, *P. parvulus* 2.6 and *L. lactis* NZ9000[pGTF] synthesize, respectively the EPS P and the EPS L polymers having the same primary structure, which is different from those of the β-glucans isolated from fungi and yeasts, which have ramifications at positions *O*-4 and *O*-6 (Thompson et al., 2010).

This peculiarity of structure was the reason why we investigated the probiotic potential of the producing strain *P. parvulus* 2.6 (Werning et al., 2012; Pérez-Ramos et al., 2017), and the prebiotic potential of its EPS P (Russo et al., 2011; Pérez-Ramos et al., 2017). Also, our previous work supports an anti-inflammatory effect of this *O*2-substituted-(1–3)-β-D-glucan. Comparison of the behavior of *P. parvulus* 2.6 with its isogenic EPS P-non-producing strain, revealed that the presence of the polymer decreased the pro-inflammatory effect exerted by the LAB on human macrophages M1, indicating a possible activity of this EPS as an immunomodulator in the innate immune response (Fernández de Palencia et al., 2009). Moreover, in an induced inflammation model, using the zebrafish transgenic line Tg(mpx:GFP)i114, the polymer inhibited neutrophil recruitment and proliferation in the larvae, confirming once again its potential as an immunomodulator (Pérez-Ramos et al., 2018). Furthermore, the metabolic rate of macrophages derived from human monocytes increased upon exposure to either the EPS P and the EPS L synthesized by *L. lactis* NZ9000[pGTF] (Notararigo et al., 2014). In addition, these EPS activated processes involved in M1 differentiation, migration, and cellular proliferation, as well as inhibited AKT and mTor pathways implicated in the inflammatory response (Notararigo et al., 2014). Therefore, this current work aimed to study the immunomodulatory effect of the *O*2-substituted-(1–3)-β-D-glucan, vs. the *O*6-substituted-(1–3)-β-D-glucan, (laminarin, isolated from *Laminaria digitata*), on ileocolonic biopsies of CD patients.

## Materials and Methods

### Patients

Samples of intestinal mucosa were obtained during surgery from four patients with CD (one male and three females, age 46.7 ± 19.9, SEM), who underwent ileal resection for stricture unresponsive to conventional medical treatment (Table 1). Biopsies of the intestinal tissue were kept at 4°C, from collection until their later handling. None of the patients had been on anti-TNF treatment for at least 2 months before the intervention.

**Table 1:** Characteristics of the patients included in the pilot study.

Patients	Age	Sex	Affected segment	Debut date	Treatment
1	20	F	Ileocecal	2 years	Azathioprine + budesonide
2	42	F	Ileocecal	less than 6 months	Amoxicillin + clavulanate
3	57	F	Ileocecal	8 years	Mesalazine + azathioprine
4	68	M	Ileocecal	28 years	Azathioprine

The diagnosis of CD was previously established by the clinical routine, as well as radiological and endoscopic, criteria, and afterwards was confirmed by histological evaluation of the surgical specimen. All patients received the same preparation for colonic surgery including gut lavage with electrolyte-polyethylene glycol solution and broad-spectrum antibiotic therapy.

The study was approved by the Ethics Committee (Comité Ético de Investigación Clínica, Hospital Vall d’Hebron, Barcelona). Written informed consent was obtained from all patients [CEIC: PR(AG)56/2010].

### EPS P and EPS L Production and Purification

Exponential cultures of *P. parvulus* 2.6 (Dueñas-Chasco et al., 1997; Pérez-Ramos et al., 2018) and *L. lactis* NZ9000[pGTF] (Werning et al., 2008) were used to produce EPS P and EPS L, respectively. The EPS were produced and purified, as previously described (Notararigo et al., 2013). Briefly, after removal of bacterial cells by centrifugation, the EPS present in the culture supernatants were precipitated with three volumes of ethanol. Then, the EPS were further purified by dialysis and fractionation by size exclusion chromatography after resuspension in a 0.3 M NaOH solution. Afterwards, the EPS alkaline solution was dialyzed as above. Finally, the EPS was lyophilized and left at room temperature until use. After the first and second lyophilization, the purity of the EPS was tested fluorometrically using specific fluorescent staining kits for DNA, RNA, and proteins as previously reported (Zarour et al., 2018). Solutions of the purified polymers were prepared at 1 mg ml^−1^. No contaminants were detected (Table 2).

**Table 2:** Detection of contaminants in EPS preparations.

Contaminants	1 lyophilization		2 lyophilization	
	EPS P	EPS L	EPS P	EPS L
DNA (%)	0	0	0	0
RNA (%)	0	<0.1	0	0
Proteins (%)	<0.1	1.2	0	0

### Organ Culture of Human Colonic Mucosa

Organ culture assays were performed as described (Borruel et al., 2002, 2003; Llopis et al., 2009). Full-thickness ileal wall specimens, including areas with macroscopic lesions, were collected at surgery. After rinsing and washing with sterile saline solution, the specimens were transferred to the laboratory in sterile saline solution at 4°C. The intestinal mucosa was removed from the tissue, and cut into pieces of approximately 25–35 mg weight each, making an equal distribution of the macroscopic lesion. Each piece was placed on the insert of a 12-well cell culture plate (Netwell culture system, Costar), with the epithelial surface uppermost. Filters were placed into the wells and incubated with 1,500 μl RPMI 1640 culture medium (CanSera), without antibiotic (37°C, 95% O_2_, 5% CO_2_ -carbogene-). Before usage, the culture medium was filtered through a 0.22 μm membrane, warmed to 37°C, and gassed for 90 min with carbogen. Tissue without lesion (not inflamed) was used as negative control.

The tissues obtained from biopsies of CD patients were exposed independently to EPS L, EPS P, or laminarin. A solution of the polymers in culture medium at 100 μg ml^−1^ was added by dripping onto the tissues. The cell culture plate was covered with a supported lid, allowing a correct exchange of gases, and subsequently placed in a bath at 37°C, inside a container shielded with a wet cloth. Then, the container was connected to the carbogen gas outlet, gassed at high pressure for 10 s at 1 h intervals, and incubated for 4 h.

Then, the pro-inflammatory (TNF-α and IL-8) and anti-inflammatory (IL-10) cytokine levels released into the media were quantified by ELISA. At the end of the experiment, aliquots of the supernatant were collected from each well, prior to being stored at −80°C, while the tissue was kept immersed in 400 μl of rRNA stabilization solution “RNA later” (Ambion) at 4°C overnight, and then stored at −80°C.

### Determination of Cytokine Levels by Elisa

The cytokine protein profiles of tissue culture supernatants, in response to EPS treatments, were analyzed by OptEIA ELISA (BD Pharmingen) to detect IL-10 and TNF-α, as well as by DuoSet ELISA (RD Systems) for IL-8, following the suppliers’ instructions.

The cytokine concentrations were extrapolated from the regression line generated using the absorbance values of a standard curve, which was produced in the same test with known concentrations of commercial cytokines.

### Gene Expression Profiling Under EPS Treatment

In gene expression organ culture assays, CD samples were harvested in the presence of RNA later (Ambion) and stored at −80°C. RNA extraction from the patients’ biopsies was performed using the RNeasy mini (Qiagen), according to the supplier’s instructions. RNA concentration and integrity were analyzed and determined with the RNA 6000 Nano Chip in a Bioanalyzer 2100 (Agilent Technologies). RNA quantity and integrity were considered acceptable if the 28S/18S ribosomal fragment ratio was over 1.5, and the RNA Integrity Number (RIN) ranged in values between 9 and 10. Selected gene expression was evaluated with real-time PCR, and 1 μg of total RNA was used for the reverse transcription to synthesize first strand cDNA following the recommended protocol of the High-Capacity cDNA Reverse Transcription kit (Applied Biosystems). Quantitative PCR was then carried out with the Taqman Gene Expression Assay (Thermo fisher Scientific), using TaqMan Fast Universal PCR Master Mix (2X; Applied Biosystems). Relative quantification of gene expression of thymic stomal lymphopietin (TSLP), IL-12p35, IL-10, and IL-8 was determined, using a 7500 Fast Real time PCR System (Applied Biosystems). Data were obtained as threshold cycle (Ct) values. Gene expression levels for each individual sample were normalized relative to the PPIA gene as housekeeping/constitutive/endogenous gene, which encodes the Peptidylprolyl Isomerase A (also called Cyclophilin A). Each condition was run in triplicate.

### Transcription Factor Signaling Pathways NF*κ*B RNA Array

The RT2Profiler PCR array (SABiosciences-Qiagen) was used to obtain the expression profile of 84 fundamental genes of the NFκB pathway (Supplementary Table S1; supplementary data).

The analysis with the array was performed according to the manufacturer’s instructions, as follows. The substrate used was cDNA synthesized from RNA samples (obtained from patient biopsies) with the “RT2 First Strand” kit (Qiagen). The quantitative-RT-PCR (qPCR) was carried out using the SYBR GREEN technology “RT2 SYBR Green,” and run in a thermal cycler iQ5 (BioRad). Once the reaction was complete, the data corresponding to the Ct values of each gene were exported to a data sheet for further analysis. The analysis of the differential gene expression was carried out through the web application developed by the company for this purpose based on the method *Δ*ΔCt and using three of the five constitutive genes for the standardization of the data (β-actine, β-2-microglobulin, and the ribosomal P0 protein; Seeger et al., 2014).

### Bioinformatic Functional Analysis

The functional analysis of the selected genes was performed using the Database for Annotation, Visualization and Integrated Discovery (DAVID 6.7; http://david.abcc.ncifcrf.gov), which integrates the information of over 1.5 million genes in more than 65.000 species, from different public sources of gene and protein notations (Huang et al., 2008). DAVID provides a functional classification of genes by extracting the information from various databases. We selected terms from Gene Ontology (GO; http://geneontology.org/) and from molecular pathways integrated in: the Biological Biochemistry Image Database (BBID, http://bbid.irp.nia.nih.gov/), Biocarta (http://www.biocarta.com/genes/index.asp), and the Kyoto Encyclopedia of Genes and Genomes (KEGG, http://www.genome.jp/kegg/).

Gene ontology terms allows the unification of the representation of the attributes of genes and their products among the species, favoring the functional interpretation of experimental data by dividing the terms into three categories: cellular compartment (CC), molecular function (MF), and biological process (BP). The use of molecular pathways databases facilitates the production of graphic information of how genes and their products interact.

### Statistical Analysis

Statistical analysis was carried out using the Prism 8 (GraphPad) software. The normality of the data was tested by the Kolmorov-Smirnov or Shapiro-Wilk Test normality test. For parametric data, a two-tailed paired *t*-test was applied, while for nonparametric data, the Friedman Test for paired data was used.

For qPCR, for parametric data, unpaired two-tailed Student *t*-test was performed with Welch’s correction. Values with *p* < 0.05 were considered significant.

## Results and Discussion

### Effect of *O*2-Substituted-(1–3)-β-D-Glucans on Cytokine Production in *ex vivo* Models

**Figure 1:**
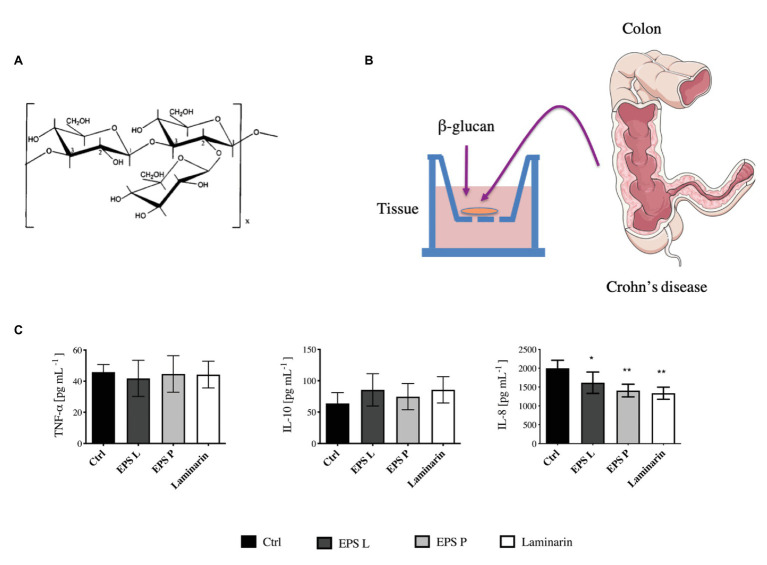
Evaluation of (1–3)-β-D-glucans in *ex vivo* model of inflamed gut mucosa. **(A)** The primary structure of EPS L and EPS M is depicted. **(B)** Organ culture assay, the tissues were collected from Crohn’s disease (CD) patients and incubated in the culture medium for 4 h. Then, the media were used to perform ELISA Immunoblot. **(C)** Concentration of tumor necrosis factor-α (TNF-α), IL-10, and IL-8 secreted by the tissues represented in Barplot mean with SEM. The levels of cytokines were quantified by the ELISA immunoblot method. Data passed the Kolmorov-Smirnov normality test, and statistical significance was tested with two tailed *t*-test between each treatment and control (Ctrl). IL-8 was decreased in inflamed tissue treated with EPS L *p* < 0.03 (^*^), EPS P *p* < 0.0068 (^**^), and laminarin *p* < 0.0097 (^**^) vs. untreated control.

The EPS P and EPS L synthesized by *P. parvulus* 2.6 and *L. lactis* NZ9000[pGTF], respectively, are identical *O*2- substituted-(1–3)-β-D-glucans with the same primary structure (Dueñas-Chasco et al., 1997; Werning et al., 2014). However, the polymers have different molecular masses, 9.6 × 10^6^ Da for EPS P and 6.6 × 10^6^ Da for EPS L (Werning et al., 2014). The molecular mass of polysaccharides plays a role in their biological activity, consequently, there may be differences between the immunomodulatory activities of each EPS, and therefore both polymers were tested. Thus, in the present pilot study, to evaluate EPS P and EPS L (Figure 1A), *ex vivo* tissue cultures have been used as a model of pathological intestinal inflammation (Figure 1B), in which the immune system plays an important role in the development, propagation, and maintenance of inflammation and disease. We have shown that this model is suitable to investigate the effects of LAB treatment on the intestinal immunity axis (Bäuerl et al., 2013). Laminarin was also tested as a positive control due to its ability to activate the immune system through its interaction with the Dectin-1 receptor (Xie et al., 2010; Smith et al., 2018).

Tissues obtained from biopsies of four CD patients were exposed independently to the polysaccharides (Figure 1B); and subsequently the pro-inflammatory (TNF-α and IL-8) and anti-inflammatory (IL-10) cytokine levels released into the media were quantified (Figure 1C). The results revealed that the concentration of the pro-inflammatory IL-8 secreted by the tissues during treatment with either of the three β-glucans tested was significantly lower (*p* < 0.05) than that released by the untreated control tissue. In addition, the highest effect was obtained with EPS P or laminarin. These results indicated a role of the *O*2-substituted-(1–3)-β-D-glucans on the reduction of inflammation associated with the intestinal epithelial mucosa. However, the levels of TNF-α and IL-10 secreted by the tissues were not affected by the treatments. This lack of influence could be due to the cytokine’s short lifespan and to the large number of proteases released by the tissue of CD patients (Borruel et al., 2002). These hypotheses presumably did not apply for the results obtained for IL-8, since the levels detected for this cytokine were 40 times higher than those observed for the TNF-α and IL-10. Therefore, the alteration of IL-8 concentration seems to reflect the biological status of the tissue. One of the CD patients, whose ileocolonic biopsy was analyzed and previously treated with antibiotic (Table 2). Therefore, the results obtained could have a bias due to the inclusion of his biopsy in the study. However, quantification of the cytokine levels released by the biopsies of only the three other patients provided the same kind of pattern and behavior (Supplementary Figure S1), validating the performed assay.

### Effect of *O*2-Substituted-(1–3)-β-D – Glucans on Gene Expression Profiling in *ex vivo* Model

**Figure 2:**
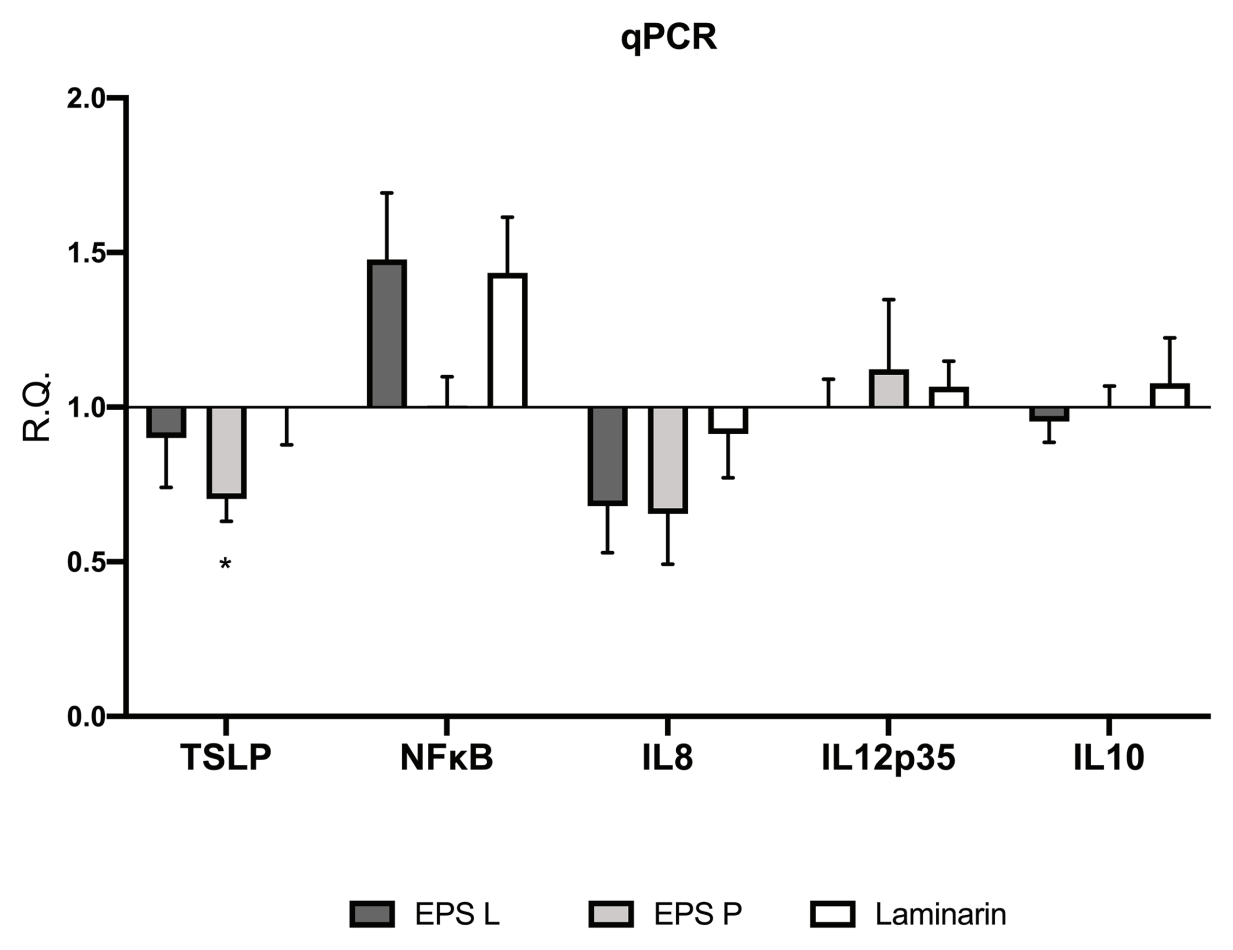
(1–3)-β-D-glucans modulate the relative gene expression of the biopsies of patients affected by CD. Barplot representation mean with SEM of TSLP, NF*κ*B, IL-8 IL-12p35, and IL-10. Gene expression was analyzed by quantitative-RT-PCR (qPCR) analysis. Data passed the Shapiro-Wilk normality test, the unpaired two-tailed Student *t*-test was performed with Welch’s correction Only TSLP demonstrated a significance difference with a *p* < 0.03 vs. untreated control.

To understand the immunomodulatory effect of the bacterial β-glucan on gene expression in the *ex vivo* model, levels of transcription of the TSLP, IL-8, IL-12p35, and NF*κ*B coding genes in the treated vs. untreated tissues were determined by means of qPCR (Figure 2).

The analysis of gene expression in the treated vs. untreated tissues revealed in general a similar pattern of response to the exposure to EPS L, EPS P, or laminarin. Thus, the treatment with any of the three β-glucans resulted in a decrease of TSLP and IL-8 transcription. However, this effect was only statistically significant (*p* = 0.0367) for TSLP in tissues treated with EPS P. TSLP is a protein, which belongs to the cytokine’s family and, that promotes T cell maturation when antigen presenting cells are activated. In IBD patients, low levels of TSLP are associated with a permeability increase of the gut barrier, or Th2 cell differentiation, depending on the cell type that is expressing it (epithelial cells or DC cells; Biancheri et al., 2016; Park et al., 2017). Recently, it has been proposed that TSLP also participate in Treg cell development and gut homeostasis perpetuation (Tahaghoghi-Hajghorbani et al., 2019). Our results suggest that EPS L and laminarin exhibit normal values of gene expression, leading to a balanced TSLP function, while EPS P slightly decreases its expression, and probably has no effect on TSLP turn over. EPS P fold change reached 0.74, which means that the decrease of relative expression is not so dramatic as expected in CD patients with active inflammatory reactions (Tahaghoghi-Hajghorbani et al., 2019).

**Figure 3:**
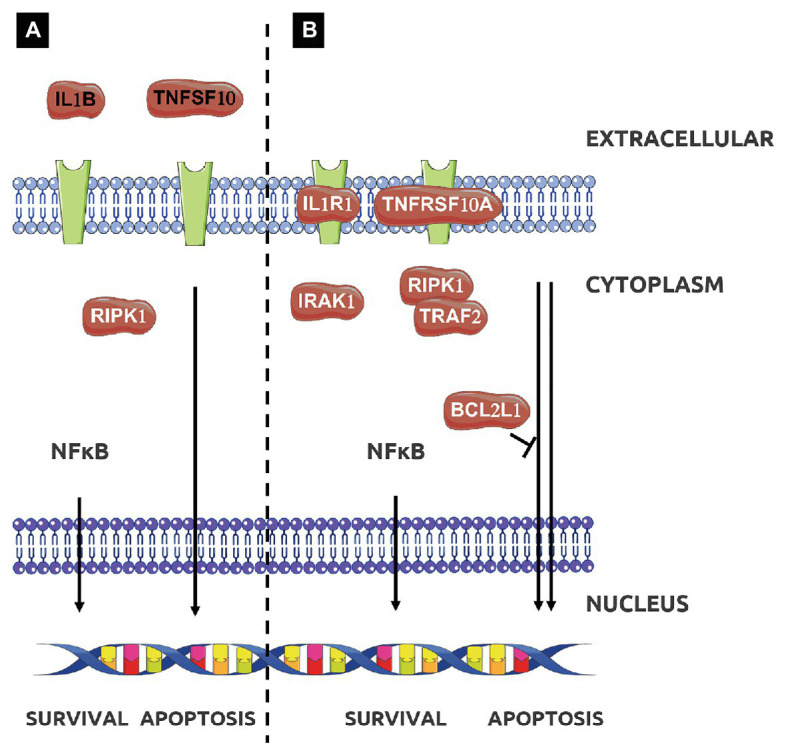
Effect of treatment with EPS L **(A,B)** or EPS P **(B)** on apoptotic signaling pathway. Adapted from Kyoto Encyclopedia of Genes and Genomes (KEGG; Kanehisa et al., 2014).

Our results also revealed that the NF*κ*B transcript was over-expressed in tissues exposed to EPS L or laminarin (Figure 2). NFκB is a transcription factor that regulates the expression of pro-inflammatory genes, including cytokines, chemokines, and other molecules. NFκB is a heterodimer formed by RelA/1 that activates the canonical signaling pathway and Rel B/NFKB2 that activates the non-canonical pathway (see details in Figure 3). The first is activated by TNF-α or IL1, while the latter is activated by cluster differentiation 40 (CD40), B cell activating factor, and other molecules, but not by TNF-α. The heterodimer activation mechanism is complex; additionally, the presence of cofactor molecules in the nucleus, that bind to the transcription factor complex, may influence the transcription of pro- or anti-inflammatory proteins coding genes (Lawrence, 2009). Its activity is regulated by the endogenous cytoplasmic inhibitors IκKs kinases complex that ensures the activation/repression of the heterodimer, that in specific conditions is able to transmigrate to the nucleus and induce transcription (Jobin and Sartor, 2000). In IBD, the canonical pathway is activated constitutively, thus conspicuous amounts of TNF-α are released by immune system cells in gut mucosa, as well as interferon-*γ* (IFN-γ) or IL-8 (Lawrence, 2009).

Our results indicate that EPS L and laminarin increased the relative expression of NFκB (RelA subunit) 1.5-fold higher than that of the control, while EPS P does not have any major effect. To comprehend the EPS effect on NFκB activation, it would be necessary to study all genes involved in the NFκB signaling pathway and clarify EPS activity. We would expect that the observed increase of NFκB expression would determine the increase of pro-inflammatory mediators, like IL-8 or TNF-α, instead we detected the reduction of the IL-8 transcript (Figure 2) and no significant difference in TNF-α detection in cultured media (Figure 1), or in gene expression (Figure 2).

IL-8 is a chemokine produced by macrophages and intestinal epithelial cells (IEC); it is considered a good marker to determine disease activity (Mohammed Vashist et al., 2018). It is also known as neutrophil chemotactic factor, because it induces chemotaxis in target cells such as neutrophils or other cells that migrate toward the infection focus (Llopis et al., 2009; Walana et al., 2018). Our results showed that the amount of the protein IL-8 (Figure 1) and the expression of its coding gene (Figure 2) decreased during treatments, suggesting that neutrophil recruitment also decreased as a consequence of the reduction of the inflammation rate. In line with these results, we have previously detected that EPS P treatment decreased IL-8 and TNF-α expression in a gnotobiotic zebrafish larvae model, indicating its ability to reduce inflammation (Pérez-Ramos et al., 2018). Also, when EPS P was tested in an induced inflammation model using the zebrafish transgenic line *Tg(mpx:GFP)i114*, a significant decrease of neutrophil recruitment to the inflammation focus was observed (Pérez-Ramos et al., 2018).

Analysis of IL12p35 gene expression confirmed that the treatments did not augment the pro-inflammatory response in the *ex vivo* model (Figure 2). The fold change values were close to the untreated control, henceforth, taking into account that IL12p35 is the specific subunit of IL12 cytokine, EPS do not activate mechanisms involved in IL12 signaling pathway. Overall, the results obtained indicated a trend toward reduction of inflammation in CD biopsies treated with any of the three β-glucans.

IL-10 is an anti-inflammatory cytokine, that is released in the gut by macrophages and IEC to maintain homeostasis (Latorre et al., 2018; Morhardt et al., 2019). According to our results, IL-10 mRNA levels did not show significant differences in the expression profiling (Figure 2), confirming the results obtained in cytokine production (Figure 1). This agrees with our previous observation that macrophages derived from human monocytes treated independently with either EPS did not stimulate IL-10 production (Notararigo et al., 2014). Moreover, we observed the same behavior in a gnotobiotic zebrafish larvae model, where EPS P treatment did not result in an increase of IL-10 expression (Pérez-Ramos et al., 2018). Taken together, these results support that bacterial β-glucans do not interact with the IL10R receptor (Shouval et al., 2014).

### Effect of EPS L and EPS P on the NF*κ*B Gene Expression Profile of CD Patient Biopsy

**Figure 4:**
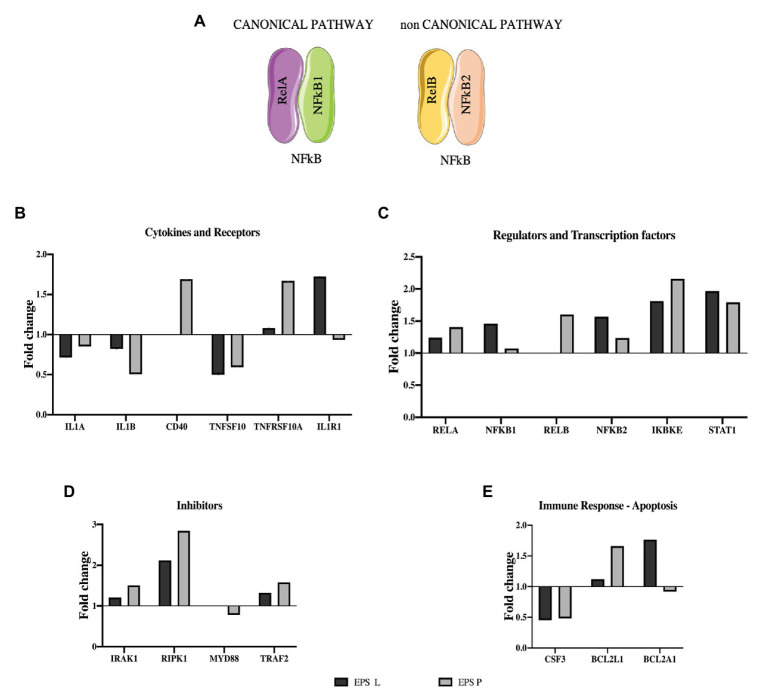
Effect of EPS P and EPS L on expression of genes involved in NFκB pathway. **(A)** NFκB heterodimers activate canonical and non-canonical pathway. **(B)**, **(C)**, **(D)**, and **(E)** Expression profiling of representative genes (fold change), whose expression resulted affected by the treatment. Data of the patient who showed the lower qPCR for IL-8.

After detecting IL-8 reduction at transcriptional and protein levels, as well as NFκB modulation, we decided to investigate the effect of β-glucans on NFκB signaling pathways, carrying out a gene expression “array” with the RNA from the CD patient that showed the greatest IL-8 relative expression reduction. Moreover, to have a representative analysis, the results obtained for EPS treated and untreated samples were normalized taking in account the expression of three houskeeping genes (see details in “Materials and Methods”), and the results are depicted in Figure 4. The profiles in response to treatment with either EPS P or EPS L were, in general similar (Figures 4B–E). The expression of the cytokines IL1A and IL1B coding genes was reduced (fold change: IL1A 0.8, 0.7; IL1B 0.5, 0.8 for EPS L and EPS P treatment, respectively) as well as the levels of transcripts encoding the colony-stimulating factor 3 (CSF3) and the MyD88 adapter protein. These results point out that both EPS have an immunomodulatory effect on the gut immune system. Receptor Interacting Serine/Threonine Kinase 1 (RIPK1) was upregulated indicating a possible function in IEC, restoring the gut barrier integrity by preventing a dysregulated cell death mechanism. Moreover, the results seemed to support that the EPS selectively activated NFκB heterodimers, EPS P RelB/NFKB2, and EPS L RelA/NFKB1 (Figure 4A; Jobin and Sartor, 2000).

We selectively studied the NFκB heterodimer complex formed by RelB/NFKB2 and RelA/NFKB1 because NFκB1 and NFκB2 act as inhibitors, blocking transcription factor translocation to the nucleus until an external stimulus activates the proteolysis that converts them into p50 and p52, respectively. The overexpression of both inhibitors, showed in Figure 4D, could be the key for the immunomodulatory effect of bacterial β-glucan.

According to these results, EPS P seemed to exert its impact mainly on the MyD88 independent signaling pathway, as shown by its influence on RelB, Interleukin 1 Receptor Associated Kinase 1 (IRAK1), RIPK1, Inhibitor of Nuclear Factor Kappa B Kinase Subunit Epsilon (IKBKE), Signal Transducer and Activator of Transcription 1 (STAT1), and CD40 (Figures 4C,D).

On the other hand, EPS L could modulate both the canonical and non-canonical pathways (Figure 4A). The first, MyD88 dependent, by activating Rel, Toll Like Receptor 6 (TLR6; data not shown), and RIPK1, and repressing IL1A and IL1B gene expression. The latter, MyD88 independent, by activation IKBKE and STAT1 (Figure 4C). In line with these findings, our previous results demonstrated that EPS P downregulated the relative expression of three genes involved in inflammation, MyD88 among them, in a zebrafish larvae model, pointing out the potential of the *O*2-substituted-(1–3)-β-D-glucan as an immunomodulator (Pérez-Ramos et al., 2018).

Although EPS P and EPS L could have a different effect on transduction pathways activated by NF*κ*B, this analysis does not fully clarify what mechanisms they may be modulating. Both have been shown to reduce the expression of pro-inflammatory cytokines, such as IL-1A, IL-1B, TNF, and IL-8 (Figure 4B), and these results have been validated in different models (Notararigo et al., 2014; Pérez-Ramos et al., 2018). The overall results were quite complex, considering that many of the genes studied are involved in the signaling of different receptors and are present in the majority of cell types isolated in the CD biopsy. For this reason, it was necessary to use bioinformatics tools to group the genes whose function may be related, and improve the understanding of the mechanisms underlying the stimulus provided by the *O*2-substituted-(1–3)-β-D-glucan, as described below.

### Functional Analysis With David Bioinformatic Resources

In order to interpret the results of the differential response caused by each EPS on the tissue of the CD patient, a bioinformatic clustering with DAVID was carry out. The aim of this analysis was to arrange genes and derive their biological meaning, by association with identical or related functions and/or pathways. We restricted gene analysis to fold changes between ≤5 and/or ≥1.5, to establish strong interaction between selected genes.

The gene clustering revealed a modulation of 14 or 19 genes upon treatment with either EPS L or EPS P. Among the regulated genes, the presence of EPS L resulted in a reduction of expression of 4, whereas downregulation of only one gene was observed with the EPS P treatment. Also, 10 or 19 where upregulated after exposure of the tissues to EPS L or EPS P, respectively. Furthermore, as a response to both treatments, the alteration of expression of five genes showed the same pattern: upregulation for IKBKE, PC4, and SFRS1 interacting protein 1 (PSIP1), RIPK1 and STAT1, and downregulation for Colony-Stimulating Factor 3 (CSF3).

The functional analysis for molecular function of GO depicted in Table 3 showed that the enrichment in functional terms for genes affected by treatment with EPS P, was greater than that with EPS L in number, degree of enrichment, and number of members in each category. In addition, EPS L regulon included genes grouped into two categories related to cytokine activity, while for EPS P regulon more grouping terms were found, highlighting the regulation of caspase activity and cytokine activity. These results confirmed the data obtained from functional grouping (data not shown), and the overall results allowed us to postulate a possible mechanism of action of either EPS on the apoptosis pathway represented in Figure 3. EPS L could affect extrinsic apoptosis pathways with the inhibition of Tumor Necrosis Factor Apoptosis-Inducing Ligand 10 (TNFSF10) and IL-1B, while also it could activate survival pathway (RIPK1), with a possible healing effect on gut barrier, through the NF*κ*B pathway and chemotaxis reduction upon CSF3 downregulation. EPS P could activate apoptosis pathway by altering gene expression of Tumor Necrosis Factor Receptor Superfamily Member 10a (TNFRSF10A), and the cell death regulator BCL2L1 while repression of CSF3 may points to a role in modulation of intestinal immunity, with a possible decrease in the chemotaxis of the neutrophils to the foci of inflammation (Carol et al., 2006).

**Table 3:** Molecular function gene ontology (GO) terms for the gene differentially expressed upon EPS P or EPS L treatment.

	GO TERM	ENRICHMENT^a^	GENES
EPS P	GO:0043028~caspase regulator activity	47,12	TNFRSF10A, BCL2L1
	GO:0005125~cytokine activity	10,51	CSF3, CCL2, and CSF1
	GO:0004674~protein serine/threonine kinase activity	7,94	IRAK1, IKBKE, MAP3K1, RIPK1, and RAF1
	GO:0004672~protein kinase activity	6,76	IRAK1, IKBKE, CCL2, MAP3K1, RIPK1, and RAF1
	GO:0008134~transcription factor binding	5,33	TNFRSF10A, RELB, BCL3, and NFKB2
	GO:0019899~enzyme binding	5,23	CSF3, IRAK1, MAP3K1, and CD40
	GO:0042802~identical protein binding	4,27	IRAK1, MAP3K1, CSF1, and BCL2L1
	GO:0003700~transcription factor activity	3,50	RELB, IRF1, BCL3, NFKB2, and STAT1
	GO:0030528~transcription regulator activity	2,71	IRAK1, RELB, IRF1, BCL3, NFKB2, and STAT1
EPS L	GO:0032813~tumor necrosis factor receptor superfamily binding	64,43	TNFSF10, RIPK1
	GO:0005125~cytokine activity	20,49	CSF3, TNFSF10, IL1B, and CCL5

a Enrichment of functional terms is shown as a proportion. It represents the gene list input divided by all the genes annotated to the selected GO term.

To support this hypothesis, some of the results depicted in Figure 4E should be discussed in the context of the apoptotic signal pathways. BCL2A1 is a gene which belongs to the BCL-2 family, the protein encoded by this gene is able to reduce the release of pro-apoptotic cytochrome c from mitochondria and block caspase activation. The gene showed a 1.7- or 0.9-fold change upon treatment with EPS L or EPS P. This gene is directly activated by NFκB in response to inflammatory mediators, such as granulocyte-macrophage colony-stimulating factor (GM-CSF), CD40, and cytokine like TNF-*α* or IL-1 which suggest lymphocyte activation/proliferation and cell survival. Yet, both EPS downregulate TNF-α, IL-1A, and IL-1B, and have no effect on GM-CSF (data not shown), while only EPS P upregulated CD40. Taken together, these results suggest that EPS L had an anti-apoptotic effect, while EPS P had a possible pro-apoptotic effect. Indeed, both β-glucans showed an anti-inflammatory effect, because of reduction of the relative expression of cytokines, that might reflect in reduced lymphocyte activation for EPS L and in cell death activation for EPS P.

The BCL2L1 gene product is a potent pro-apoptotic activator, and this gene was upregulated by both EPS treatments with a fold change of 1.6 for EPS P and 1.2 for EPS L. We postulate that the *O*2-substituted-(1–3)-β-D-glucans might reestablish normal condition of the intestinal immune system cells through an apoptosis activation mechanism (Figure 3B).

In addition, expression of the cytokine TNFSF10 was downregulated in the presence of both EPS (fold change: 0.5 and 0.4 for EPS P and EPS L treatments, respectively) while TNFRSF10A, that binds TNFSF10 was slightly upregulated under EPS P treatment (fold change 1.3), and downregulated upon exposure to EPS L (fold change: 0.7). Moreover, Tumor Necrosis Factor Receptor Superfamily Member 10b (TNFRSF10B), and IL-1R receptors coding genes were downregulated (fold change: 0.9 and 0.7, respectively) in EPS L treated tissues, and upregulated (fold change: 1.4 and 1.7, respectively) in tissues exposed to EPS P (Figures 4B,C). These results indicate that EPS L and EPS P reduced the expression of the apoptotic ligand TNFSF10, but had a different effect on the expression of receptor TNFRSF10B, an IL-1R. It is possible that EPS P activates a cell death mechanism, to regulate lymphocyte proliferation, while EPS L acts at the cell mediation level by reducing cytokine production.

Therefore, EPS P seems to modulate apoptosis, as several results previously collected support the bioinformatic analysis. Some examples are, the lowering of IL-8 in an *ex vivo* model, a zebra fish model and in an *in vitro* transwell model (unpublished data). The decreased level of IL-1B and IL12p35 in LPMCs isolated from CD biopsies and treated with either EPS, detected with Luminex (data not shown). IL-8 takes part in the neutrophil recruitment, while IL-1B and IL-12p35 are Th1 mediators that augment when Th1 cells are deregulated, therefore indicating a possible implication of physiological modulation of apoptosis in these T cells subpopulations. It is well-established that IBD pathogeneses determines Th1/Th2 imbalance toward Th1 (Neurath, 2014). Th1 proliferation is decontrolled because apoptosis regulation is imbalanced, increasing inflammation and chemotaxis in the gut. Hence, Th1 cells overproduce pro-inflammatory mediators like: TNF-α, IL1A, IL1B, IFN-*γ* etc. In turn TNF-α, that has a pivotal role in the mucosal inflammation, recruit, and over stimulate immune system cell like Th1.

Our data suggest that apoptosis intrinsic pathway mechanism (Stevens and Oltean, 2019), or in ameliorating gut barrier inflammation (Lu et al., 2020), could be re-established after *O*2-substituted-(1–3)-β-D-glucan administration, due to the fact that Bcl2 genes are overexpressed (Figure 4). According to that, Th1 proliferation could be regulated and therefore reducing proinflammatory cytokine production’s (Figures 1C, 2, 4E).

As IBD is Th1 shifted pathology, and EPS P is able to ameliorate the rate of inflammation, it is plausible to postulate that it could play a role in Th1 through apoptosis modulation. Bioinformatic analysis revealed that many mediators of this pathway have been modulated by the treatment (Table 3), and we also observed a reduction of pro-inflammatory mediators (Figures 1C, 2). As the Th1 of CD patients cycles faster than the healthy controls (Sturm et al., 2004), it is fair to believe that Th1 could play a role in the re-establishment of Th1 population to a normal concentration, and therefore lowering inflammation rate.

Regarding EPS L, gene clustering showed a dual behavior, by the reduction of cell death ligands expression such as TNFSF10 and IL-1B, and over-expression of RIPK, a kinase involved in necroptosis and cell survival, by modulating dysregulated apoptosis in IEC (Figure 3A; Dannappel et al., 2014). Thus, the EPS treatments affect mechanisms that restore gut burrier homeostasis due to its wound healing properties and reduce inflammation mediators.

It is worth noting that the differential response of the tissues to EPS P and EPS L might be related to their structural differences. EPS P has a molecular mass of 9.6 × 10^6^ Da, higher than that of EPS L (6.6 × 10^6^ Da, Werning et al., 2014), that could increase the affinity of the natural *O*2-substituted-(1–3)-*β*-D-glucan (EPS P) affinity for its receptor(s). It has been shown that molecular mass and tertiary structure of β-glucans, play a critical role in receptor binding (Legentil et al., 2015). We have previously shown that EPS P increases adhesion of *P. parvulus* 2.6 to human enterocytes *in vitro* and increases *in vivo* the colonization capacity of the bacterial strain in the zebrafish gut (Pérez-Ramos et al., 2018). Therefore, it is tempting to assume that, after ingestion in a beverage, *P. parvulus* 2.6 could synthesize EPS P in the human intestine and the action of glycosidases synthesized by the microbiota could reduce its molecular mass generating an EPS L-like polymer with its detected dual role.

## Conclusion

In an *ex vivo* model of CD biopsies, treatment with EPS P and EPS L are concomitant with decreased levels of pro-inflammatory cytokine IL-8 in the supernatant of tissue biopsies, and in relative gene expression. RNA array demonstrated that *O*2-substituted-(1–3)-β-D-glucans modulate the NFkB pathway, activating both the canonical and non-canonical pathway. The differences found in the expression profiling indicate that the 9.6 × 10^6^ Da EPS P may be able to restore the apoptosis mechanism in Th1 cells, whereas the 6.6 × 10^6^ Da EPS L might restore the intestinal barrier through activation of RIPK1 in IEC. Further studies have to be performed to determine which receptor is activated by EPS P and EPS L and, if a co-receptor is necessary for receptor recognition, and if EPS have different effects on immune system cells.

## Data Availability Statement

The original contributions presented in the study are included in the article/Supplementary Material, further inquiries can be directed to the corresponding authors.

## Ethics Statement

The studies involving human participants were reviewed and approved by Comité Ético de Investigación Clínica, Hospital Vall d’Hebron, Barcelona. The patients/participants provided their written informed consent to participate in this study.

## Author Contributions

SN performed all the experiments described in this article, realized the statistical analysis, and wrote the manuscript. EV and AO helped with the *ex vivo* model during SN stay at Vall d’Hebron laboratory. IC realized the bioinformatic analysis and with the design of the Figures. MA supervised the *ex vivo* model and the RNA Array. FG reviewed the results of the manuscript. PL and AP masterminded the direction and conceptualization of the study, the interpretation of the data obtained, and the final version of the manuscript. All authors listed have read and approved the final version of the manuscript.

### Conflict of Interest

The authors declare that the research was conducted in the absence of any commercial or financial relationships that could be construed as a potential conflict of interest.
