# Supplementary material for: Evaluation of an O2-Substituted (1–3)-β-D-Glucan, Produced by Pediococcus parvulus 2.6, in ex vivo Models of Crohn’s Disease
Source: Front Microbiol. 2021 Feb 5;12:621280. doi: 10.3389/fmicb.2021.621280 (PMC7893136; doi:10.3389/fmicb.2021.621280)
Supplement: Supplementary file 1 [file Data_Sheet_1.pdf]

Evaluation in *ex-vivo* models of patients affected by Crohn disease of the *O*2-substituted (1-3)-β-D-glucan produced by *Pediococcus parvulus* 2.6

Sara Notararigo[1,2,3], Encarnación Varela[2,4], Anna Otal[2], Iván Cristobo[1], María Antolín[2], Francisco Guarner[2], Alicia Prieto[1*], Paloma López[1*]

[1]Department of Microbial: and Plant Biotechnology, Margarita Salas Biological Research Centre (CIB-Margarita Salas-CSIC). Madrid, Spain

[2]Department of Gastroenterology, Digestive System Research Unit, Institut de Recerca Vall d'Hebron (VHIR), University Hospital Vall d'Hebron, Universitat Autònoma de Barcelona, Barcelona, CIBER EHD, Instituto de Salud Carlos III, Spain

[3]Foundation Health Research Institute of Santiago de Compostela (FIDIS), Santiago de Compostela Spain.

[4]CIBERehd, Instituto de Salud Carlos III, Madrid, Spain

Supplementary Table *S*1. NFκB signaling pathway genes analyzed with "RT^2 Profiler PCR array".

NFκB PATHWAY
**LIGANDS AND RECEPTORS**
CD27 (TNFRSF7), CD40 (TNFRS5), EGFR, F2R, FASLG (TNFSF6), IL10, IL1A, IL1B, IL1R, IL8, LTBR, NOD1 (CARD4), TLR1, TLR2, TLR3, TLR4, TLR6, TLR9, TNF, TNFRSF1A, TNFRSF10A, TNFRSF10B, TNFSF10, TNFSF14
**INHIBITORS**
BIRC2 (c-IAP2), FADD, IRAK1, IRAK2, IRF1, MYD88, RIPK1, TBK1, TICAM1 (TRIF), TICAM2, TNFAIP3, TRADD, TRAF2, TRAF3, TRAF6
**NFκB CYTOPLASMIC REGULATING MOLECULES**
BCL3, CHUK (IKKa), IKBKB, IKBKE, IKBKG, NFKBIA, NFKBIB (TRIP9), NFKBIE
**TRANSCRIPTION FACTORS**
NFKB1, NFBB2, REL, RELA, RELB
**GENES ACTIVATED BY NFκB**
**IMMUNE RESPONSE**
CCL2 (MPC-1), CCL5 (RANTES), CSF1 (MCSF), CSF2 (GM-CSF), ICAM1, IFNA1, IFNG, IL8, LTA (TNFB), TNF
**APOPTOSIS**
AGT, BCL2A1 (BCL-X), BCL2L1, BIRC3 (c-IAP1)
**OTHER NFκB SIGNALING FACTORS**
**KINASES**
AKT1, MAP3K1, RAF1
**TRANSCRIPTION FACTORS**
ATF1, EGR1, ELK1, FOS, JUN, STAT1
**OTHER GENES**
BCL10, CARD11, CASP1 (ICE), CASP8, CFLAR (CASPER), HMOX1, MALT1, PSIP1, RHOA, TIMP

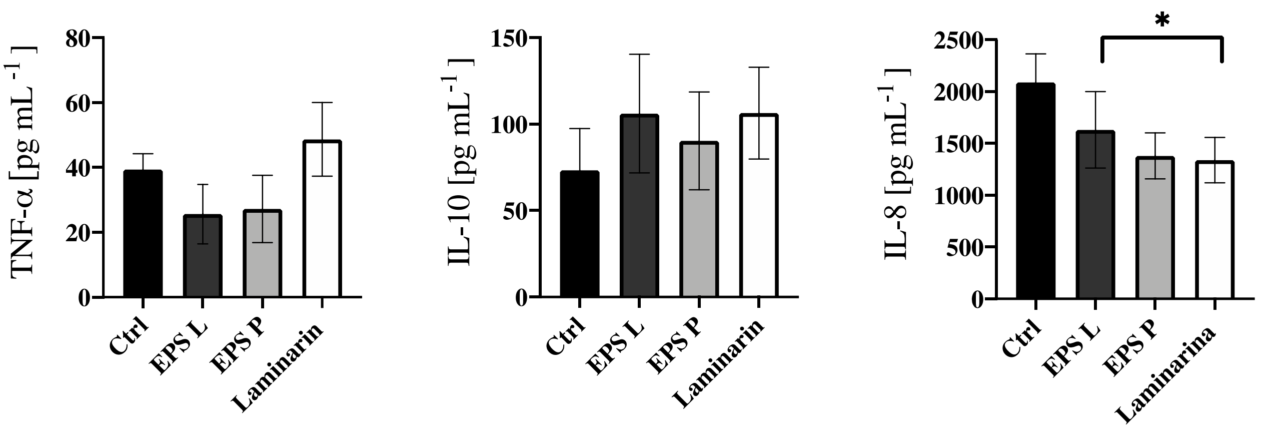

Figure S1: Organ culture cytokine production of 3 patients included in the study and not treated with antibiotics. Box whiskers representation with mean values and SEM. Data passed the normality KS test, then two tailed t test was performed for parametric paired values. Only IL-8 secretion was significant with a $p < 0.04$ for EPS L, $p < 0.01$ for EPS P and $p < 0.01$ for laminarin. No significant different were found for TNF-α and IL-10.
